# The Bright Side of Hospital Life.—A Rejoinder

**Published:** 1888-05-12

**Authors:** 


### THE BRIGHT SIDE OF HOSPITAL LIFE.—A REJOINDER.

IN answer to "E. L." wishing you would speak strongly about the rough side of hospital life for nurses, I should like you to speak of the bright side of it. I have been engaged in hospital work for the last ten years, two of them being spent in a workhouse infirmary, and I must say I have never found a dark side to a true nurse's life yet. As for the living and time given for meals, I can only speak in the highest terms. If "E. L." would just for twelve months make it her duty to look on the bright side of a nurse's life, and try to improve every place she goes to, she would have very little to complain of.

A NURSE OF THE SAMARITAN FREE HOSPITAL.

